# Pre-hospital antibiotic treatment and mortality caused by invasive meningococcal disease, adjusting for indication bias

**DOI:** 10.1186/1471-2458-9-95

**Published:** 2009-04-03

**Authors:** Emilio Perea-Milla, Julián Olalla, Emilio Sánchez-Cantalejo, Francisco Martos, Petra Matute-Cruz, Guadalupe Carmona-López, Yolanda Fornieles, Aurelio Cayuela, Javier García-Alegría, the ANCA Group

**Affiliations:** 1 Hospital Costa del Sol, Marbella, Spain; 2 Escuela Andaluza de Salud Pública, Granada, Spain; 3 Facultad de Medicina, Universidad de Málaga, Málaga, Spain; 4 Dirección General de Salud Pública, Tenerife, Spain; 5 Hospital Virgen del Rocío, Seville, Spain; 6 CIBER Epidemiología y Salud Pública (CIBERESP), Barcelona, Spain

## Abstract

**Background:**

Mortality from invasive meningococcal disease (IMD) has remained stable over the last thirty years and it is unclear whether pre-hospital antibiotherapy actually produces a decrease in this mortality. Our aim was to examine whether pre-hospital oral antibiotherapy reduces mortality from IMD, adjusting for indication bias.

**Methods:**

A retrospective analysis was made of clinical reports of all patients (n = 848) diagnosed with IMD from 1995 to 2000 in Andalusia and the Canary Islands, Spain, and of the relationship between the use of pre-hospital oral antibiotherapy and mortality. Indication bias was controlled for by the propensity score technique, and a multivariate analysis was performed to determine the probability of each patient receiving antibiotics, according to the symptoms identified before admission. Data on in-hospital death, use of antibiotics and demographic variables were collected. A logistic regression analysis was then carried out, using death as the dependent variable, and pre-hospital antibiotic use, age, time from onset of symptoms to parenteral antibiotics and the propensity score as independent variables.

**Results:**

Data were recorded on 848 patients, 49 (5.72%) of whom died. Of the total number of patients, 226 had received oral antibiotics before admission, mainly betalactams during the previous 48 hours. After adjusting the association between the use of antibiotics and death for age, time between onset of symptoms and in-hospital antibiotic treatment, pre-hospital oral antibiotherapy remained a significant protective factor (Odds Ratio for death 0.37, 95% confidence interval 0.15–0.93).

**Conclusion:**

Pre-hospital oral antibiotherapy appears to reduce IMD mortality.

## Background

Invasive Meningococcal Disease (IMD) remains an important cause of morbidity and mortality worldwide [1]. Although in-hospital antibiotic use and intensive care support have become more widespread, the mortality associated with IMD has remained stable over the last thirty years [2]. Pre-hospital antibiotic therapy has been recommended to lower the incidence of IMD-associated mortality [3,4], but an important controversy remains. A recent systematic review of the literature showed that indication bias was present in all the studies published, and therefore no definitive advice could be given concerning this medication [5]. Indication bias was present in outpatients receiving parenteral antibiotics and in those receiving oral antibiotherapy. In the former case, the patients with a poor prognosis are probably those who receive pre-hospital parenteral antibiotics [6,7], and the benefit of the intervention is hard to demonstrate. In the second group (patients receiving pre-hospital oral antibiotics), the patients with a better *a priori *prognosis are probably given oral antibiotics; they are less likely to have experienced an explosive clinical course and tend to present fewer alarm symptoms. In this case, thus, antibiotics are being given to those with less severe forms of IMD (indication bias), and so the actual effect of the intervention cannot be postulated.

A randomised, clinical trial to clarify the repercussion of the use of oral antibiotics within the context of IMD is difficult to design, as the disease generally requires a very high index of clinical suspicion to be diagnosed at the onset of the process.

In this study we attempted to analyse the effect of pre-hospital oral antibiotics, while controlling for indication bias through a statistical technique called the propensity score method. Propensity score was proposed in 1983 as a technique to control the *a priori *probability of receiving one or another treatment [8], attempting to equate it with randomisation in circumstances where this is not possible. In this study, the propensity score was used to assign each patient the possibility of pre-hospital oral antibiotherapy in accordance with symptoms registered before hospital admission, thereby seeking to relate the likelihood of receiving this treatment to the symptoms recorded in the clinical history – these being the reason for the antibiotherapy. Recording the objective data found on examination, for all the patients from the onset of symptoms, is not possible. However, it is feasible to identify the symptoms recorded in a clinical history and to determine the possible association of any symptom or symptoms with a better or worse prognosis of IMD.

## Methods

### Design

A retrospective follow-up study was conducted from week 40 in 1995 to week 41 in 2000 at 31 hospitals in Andalusia and the Canary Islands (Spain). The study was approved by the Ethics and Research Committee of the Costa del Sol Hospital (Marbella, Spain).

### Patients

All cases of IMD, coded as such on patient discharge reports in the diagnostic registries at each hospital, were collected during the study period. Eligible patients were all those diagnosed with IMD and aged one year or older. The diagnosis was interpreted as **definite **if there was a microbiological culture of *Neisseria meningitidis *from a sterile sample, **probable **if this condition was not fulfilled but there was a Gram stain compatible with *Neisseria meningitidis*, and **possible **if neither of these two conditions were fulfilled but the diagnosis was based on clinical suspicion.

### Variables and Measurements

The following variables were recorded: demographic data (sex, age); number of contacts with Health Services at which the patient presented similar symptoms to those leading to hospital admission; symptoms prior to admission; physical signs on admission to the emergency department, including axilar temperature, heart rate and systolic blood pressure; use of oral antibiotics during the two weeks before admission; time from onset of symptoms to administration of parenteral betalactams; and status at discharge. Microbiological data collected included the results of blood cultures and/or cerebrospinal fluid (CSF) and a Gram stain.

### Statistical Analysis

We estimated that at least 1,103 patients would be needed for the study to achieve 80% power to detect an odds ratio (OR) of 2, assuming a 30% prevalence of out-patient antibiotic use, a mortality rate of 5% in persons not using antibiotics and a two-sided α of 5%. The primary end point was mortality during hospital stay. The mean and 95% confidence interval (CI) were calculated for the quantitative variables, and percentages were calculated for the qualitative variables. A univariate analysis was performed for the result variable "death", to estimate the OR and the corresponding 95% CI.

The propensity score was defined as the probability of receiving pre-hospital oral antibiotics according to the symptoms present prior to admission. The symptoms were selected in accordance with the natural progression of IMD and, thus, with the decision to prescribe antibiotics or not. To estimate this probability, an initial logistic regression analysis was performed with all the symptoms as independent variables and the use of antibiotherapy (yes/no) as the dependent variable, using P < 0.05 as the inclusion criterion for independent variables. Further logistic regression analysis was then performed with the propensity score, out-patient oral antibiotics, time from onset of symptoms to parenteral antibiotics and age as independent variables and mortality as the dependent variable. The pre-hospital use of antibiotherapy and the variable propensity score were obligatory components in the model, with the other variables being included if they fulfilled the statistical criterion (P < 0.05). This analysis was then repeated excluding clinical suspicions. For all these analyses, SPSS 10.0 statistical software was used. After calculating the propensity score for the patients, each of those who had received extra-hospital antibiotherapy was matched with a control who had not – specifically, with the patient presenting the closest propensity score – using R 2.4.1 statistical software. This 1:1 pairing gave rise to a loss of patients who had not been exposed to extra-hospital antibiotherapy. From these two groups of patients, balanced as regards their possibility of receiving extra-hospital antibiotherapy, we compared the proportion of deaths, again using the χ^2 ^test or Fisher's exact test when appropriate, thus generating a new OR with its corresponding 95% CI.

## Results

### Description of the cohort and univariate association with death

A total of 848 patients were studied, 49 (5.72%) of whom died. The mean age of the cohort, 449 (52.9%) of whom were male, was 10.4 years (95% CI, 9.4–11.3). The older the patient, the greater the mortality, with 4.9% deaths in patients aged under 11 years vs. 25% deaths in those over 65 years.

The number of health service contacts prior to admission was zero in 502 patients (59.2%) and one in 269 (31.7%). No difference in mortality was observed depending on the number of health service contacts. No deaths occurred among those who had three or more contacts prior to admission.

Table 1 shows the symptoms recorded in the clinical history, with the corresponding OR exploring the association with mortality. The most frequent symptom was fever, while convulsions were the first symptom to appear on admission. More specific symptoms of IMD, such as petechiae, occurred in just over half the patients, and had a mean duration of eight hours before admission. The presence of convulsions and cold-like symptoms was significantly associated with higher mortality, whereas the presence of nausea/vomiting was a protective factor. The physical signs and symptoms on admission to hospital are shown in Table 2. Again, the most frequent symptom was fever, followed by petechiae.

**Table 1:** Symptoms recorded on clinical history, with OR for death (univariate analysis).

**Symptom**	**N (%)**	**Mean duration in hours (95% CI)**	**OR (95% CI)**
Fever	814 (96)	22 (21–23)	0.44 (0.14–1.30)
Nausea/Vomiting	647 (76.3)	18 (16–19)	0.47 (0.26–0.85)
Petechiae	432 (50.9)	8 (6–9)	0.77 (0.43–1.38)
Headache	338 (39.9)	23 (21–26)	0.72 (0.39–1.33)
Low level of consciousness	334 (39.4)	15 (13–17)	0.60 (0.31–1.13)
Cold-like symptoms	127 (15)	60 (48–72)	2.43 (1.27–4.66)
Pharyngoamygdalitis	119 (14)	35 (29–41)	1.2 (0.55–2.65)
Arthralgia	113 (13.3)	20 (16–25)	1.29 (0.58–2.83)
Neck stiffness	71 (8.4)	16 (10–22)	0.45 (0.11–1.89)
Irritability	50 (5.9)	14 (11–18)	0.32 (0.04–2.36)
Convulsions	37 (4.4)	3 (1–4)	2.72 (1.01–7.33)

**Table 2:** Physical signs on the initial examination at admission to the emergency room, with OR for death (univariate analysis).

**Physical sign**	**N (%)**	**OR (95% CI)**
Fever	621 (73.2)	0.82 (0.44–1.53)
Petechiae	562 (66.3)	0.72 (0.40–1.30)
Kernig/Brudszinky signs	405 (47.8)	0.68 (0.37–1.22)
Nausea/Vomiting	282 (33.3)	0.64 (0.33–1.24)
Pharyngoamygdalitis	259 (30.5)	0.3 (0.13–0.71)
Low level of consciousness	234 (27.6)	1.89 (1.04–3.4)
Cyanosis	117 (13.8)	2.41 (1.24–4.7)
Irritability	110 (13)	0.93 (0.39–2.24)
Cold-like symptoms	75 (8.8)	0.66 (0.20–2.17)
Arthritis-arthralgia	61 (7.2)	0.53 (0.12–2.25)
Convulsions	20 (2.4)	4.35 (1.40–13.55)

The mean axilar temperature, measured in the emergency room in 678 patients (80%), was 38.2°C (95% CI, 38.2–38.3). The average heart rate, measured in 519 patients (61.2%), was 128 beats per minute (95% CI, 125–130) and the mean systolic blood pressure, measured in 572 patients (67.5%), was 102.6 mmHg (95% CI, 100.8–104.5). An axilar temperature ≥40°C, a heart rate ≤60 bpm or a systolic blood pressure ≤80 mmHg were associated with higher mortality (OR for each: 4, 95% CI, 1.1–14.3; 20.7, 95% CI, 4.4–96.5; and 2.7, 95% CI, 1.3–5.6, respectively). Of the cases examined, 75.5% were definite, 4.6% were probable and 19.9% were possible.

Of the entire cohort, 226 patients (26.7%) received pre-hospital antibiotics. The main characteristics of the patients, grouped according to whether they had received pre-hospital antibiotics or not, are shown in Table 3. The mortality among the patients who had taken antibiotics was 2.7% vs. 6.9% among those who had not (OR: 0.37, 95% CI: 0.15–0.88). The main group of antibiotics used was betalactams (181 patients), followed by macrolides (36 patients). The vast majority of patients (92.2%) had begun antibiotics during the 48 hours prior to hospital admission. The main point of indication for antibiotic therapy was the primary care physician (48.2%), followed by hospital doctors (26.8%). 10.5% of the patients who took antibiotics had self-medicated. The mean time from the first symptoms to the first dose of parenteral antibiotic in the hospital was 29.7 h. No difference was observed in the time from the first symptoms to the first dose of parenteral antibiotic between those who had taken antibiotics prior to arrival at the hospital and those who had not.

**Table 3:** Characteristics of patients according to whether they received pre-hospital antibiotic treatment or not.

**Variable**	**Pre-hospital antibiotic use**	**No pre-hospital antibiotic use**
Gender (% male)	50.4	53.8
Age, mean (years)	9.6	10.2
Fever	95.6	96.1
Nausea/Vomiting	82.3	74.1
Petechiae	46.5	52.6
Headache	45.6	37.8
Low level of consciousness	39.8	39.2
Cold-like symptoms	16.8	14.3
Pharyngoamygdalitis	30.5	8
Arthralgia	11.9	13.8
Neck stiffness	13.3	6.6
Irritability	3.1	6.9
Convulsions	3.5	4.7
Time first symptoms to in-hospital antibiotics, mean (hours)	38.5	26.6
Definite cases	77.9	89.1

Categoric variables, such as symptoms, are given in percentages

A total of 323 patients (38.1%) had sepsis, 336 (39.6%) meningitis and the rest a mixed clinical form, with mortality rates of 10.7%, 2.1% and 4.2%, respectively (P < 0.001). Sepsis vs. the other clinical forms had an OR for death of 4 (2.2–7.6).

### Logistic regression analysis

The regression model used to estimate the probability of the indication for pre-hospital antibiotic use included all of the symptoms listed in Table 1. Logistic regression analysis was performed to control the association between pre-hospital antibiotherapy and in-hospital mortality, adjusting for propensity score, time from first symptoms to first dose of parenteral antibiotic in hospital and age. The clinical forms variable was excluded because it presented colinearity with symptoms. In this analysis, only age and the use of antibiotics were significantly associated with death. The OR for age was 1.03 (95% CI, 1.01–1.04) and for the use of antibiotics 0.37 (95% CI, 0.15–0.93) (Table 4). No other variable had a significant association. Thus, the OR for death in patients who did not take antibiotics was 2.7 (95% CI, 1.07–6.66) and the number needed to treat (NNT) to avoid a death was 23.5 (95% CI, 14–73.2).

**Table 4:** Results of logistic regression analysis, with death as dependent variable.

**Variable**	**OR**	**95% CI**
Propensity score	0.95	0.09–9.76
Pre-hospital antibiotic use	0.37	0.15–0.93
Age	1.03	1.01–1.04
Interval symptoms to in-hospital treatment	0.99	0.98–1.01

When the logistic regression analysis was performed with the cohort, and clinical suspicions were excluded, age continued to present a significant association with death (OR 1.03; 95% CI, 1.01–1.05) but this was not so for the use of antibiotics (OR 0.4; 95% CI, 0.11–1.4).

A further analysis was then performed. Cases of patients who had received pre-hospital antibiotic treatment were matched with those of other patients who had not, using the closest propensity score value to determine this matching. Tables 5 shows the effects of this matching, on baseline age values, symptoms and time from onset of first symptoms to first in-hospital dose of parenteral antibiotics. A sub-analysis of 436 patients (218 in each arm) was then performed to examine the association between pre-hospital antibiotic use and death, producing an OR of 0.41 (95% CI, 0.13–1.17) for antibiotic use.

**Table 5:** Mean values of the variables included in the matching process, before and after the latter was performed.

**Variable**	**Mean value** **(treated patients) before matching**	**Mean value** **(non-treated patients) before matching**	**Mean value** **(treated patients) after matching**	**Mean value** **(non-treated patients) after matching**
Age (years)	9.6	10.2	9.6	9.5
Fever	0.05	0.04	0.04	0.04
Nausea/Vomiting	0.54	0.62	0.54	0.55
Petechiae	0.16	0.25	0.16	0.16
Headache	0.88	0.86	0.88	0.88
Reduced LC	0.59	0.86	0.59	0.58
Cold-like symptoms	0.97	0.92	0.96	0.96
Pharyngoamygdalitis	0.83	0.86	0.83	0.80
Arthralgia	0.70	0.92	0.70	0.78
Neck stiffness	0.87	0.93	0.86	0.84
Irritability	0.96	0.95	0.96	0.95
Convulsions	0.55	0.47	0.55	0.56
STI (hours)	38.55	26.62	38.5	35.8

LC: Level of consciousness; STI: Symptom-treatment interval.

## Discussion

The use of oral antibiotherapy prior to hospital admission has resulted in significant benefits in terms of reduced mortality. It is evident that the use of antibiotics diminishes the mass of *Neisseria meningitidis*, and that the production of cytokines and endotoxins is generally lower in patients taking pre-hospital antibiotics [9]. Previous studies have shown that the use of pre-hospital antibiotics is associated with less culture positivity (supporting the hypothesis of a greater reduction in bacterial mass) and a lower rate of clinical complications [10].

Our study began before the large-scale vaccination of children against *Nesseria meningitidis *but the effect of antibiotics later on in life is very probably the same now, because the protection acquired is independent of serogroup. On the other hand, mortality during outbreaks is greater than during an endemic situation [11]. Spain was suffering an outbreak when cases for this study were being collected. We cannot therefore affirm that the use of pre-hospital oral antibiotherapy is beneficial in non-outbreak conditions. No patient presented evidence of pre-hospital parenteral antibiotic use, and so oral use alone (collected in the clinical report in all cases) was assumed.

An important group of patients not included in this study is constituted of those diagnosed with IMD and who died before hospital admission. This, obviously, is a special group and we cannot assess the efficacy of their treatment. Another possible source of confusion concerns the patients with suspected IMD, as symptoms could pertain to diseases other than IMD. However, the logistic regression results for the entire cohort and for the cohort excluding these patients were similar. Another limitation of our study is that memory bias may be present in the clinical report: the family or the patients may not have recalled all the symptoms or whether pre-hospital antibiotics were taken. However, this bias is compensated for by the fact that the cases were collected during a time of great fear of IMD in Spain, with an important diffusion of information in the mass media.

To date, only five cohort studies have been published exploring the relationship between the use of oral antibiotics and mortality for IMD [7,12-15]. Except for the cohort analysed by Barquet *et al*., no other study has controlled for the relationship between use of antibiotics and death for any covariable. Although all the studies found benefits to be gained from oral antibiotics, some authors, such as Morant, advise against their use, on the grounds that this treatment reduces the possibility of obtaining an accurate microbiological diagnosis and that the benefit in terms of survival is not significant. Indication bias can be observed in the work of García *et al*., in which all the patients who received oral antibiotherapy presented meningitis as a clinical form on admission to hospital. Only the study by Barquet *et al*. found a significant association between oral antibiotics and mortality. This association was controlled for age, neurological focus and haemorrhagic diathesis. Of these groups, two could be inferred by the use of antibiotics (neurological focus and haemorrhagic diathesis). In other words, pre-hospital antibiotics could, theoretically, have influenced the appearance or otherwise of these two symptoms; thus, the use of clinical items present at admission to hospital is not a good way of controlling the previous use of antibiotherapy. As the use of antibiotics may well modify the progression and prognosis of IMD, it is preferable to control the relationship between antibiotics and mortality through a variable that is less affected by treatment. Accordingly, we built our propensity score using symptoms studied in the anamnesis. This explains why the antibiotics prescribed and the mortality rate were less influential than the data determined in the emergency room. The other three studies did not control for the effect of antibiotics on mortality caused by IMD. The comments to the studies with oral antibiotics were uniform, and we are unaware if treatment was given to a group of patients who were healthier than those not taking antibiotics, an observation that has also been made in a recent systematic review of the literature [5].

It is difficult to elucidate the actual value of pre-hospital antibiotics in reducing mortality from IMD. One solution might be to perform a randomised clinical trial. However, IMD is a very difficult disease to diagnose prior to hospital admission. Only a large cohort study including cases diagnosed at all levels of the Health Service could answer this question. The use of statistical techniques such as the propensity score to control for indication bias could be useful. Such techniques enable us to control for the known risk factors reasonably well, but not the unknown ones; therefore, we must be cautious in the final interpretation. Another important concern is the relationship between pre-hospital parenteral antibiotics and death from IMD, which is not studied in this paper.

At a time when patients who receive in-hospital antibiotics generally respond well, and when support treatment has improved and large-scale vaccination programmes have been implemented, the pre-hospital use of antibiotics remains one of the few treatments remaining to be implemented in order to improve survival rates among patients with IMD, and then it may no longer be repeated that "no infection kills so quickly" [16].

## Conclusion

Pre-hospital oral antibiotherapy appears to reduce IMD mortality, controlling indication bias.

## Competing interests

The authors declare that they have no competing interests.

## Authors' contributions

Conception and design EPM, JO, GC, PM, YF, AC. Revision of the different versions of the study protocol EPM, AC. Collection and assembly of data EPM and the ANCA Group*. Quality control of the data EPM, JO. Analysis and interpretation of the data EPM, JO, ESC, FM. Drafting of the article JO. Critical revision of the article for important intellectual contents JGA. Final approval of the article JO, EPM.

## Pre-publication history

The pre-publication history for this paper can be accessed here:


